# Extending an Almost Complete Pair of Matrices to a Complete Triple

**DOI:** 10.1155/2014/493606

**Published:** 2014-04-27

**Authors:** Sheng Chen, Chao Xia

**Affiliations:** Department of Mathematics, Harbin Institute of Technology, Harbin 150001, China

## Abstract

Motivated by the concept of complete pairs, which was introduced by Krein and Langer, we present the concepts of an almost complete pair of matrices and a complete triple of matrices. It is proved that an almost complete pair of matrices can be extended to a complete triple. An application of the result to differential equations is also given.

## 1. Introduction

Let

(1) $$\begin{array}{c} L\left(\lambda \right)=I\lambda^{l}+A_{l-1}\lambda^{l-1}+\cdots +A_{0} \end{array}$$

be a monic matrix polynomial, where *A*_*i*_ ∈ *M*_*n*_(*C*) for *i* = 0,1,…, *l* − 1. If there exist a matrix *S* and a polynomial *L*_1_(*λ*) of degree *l* − 1 such that *L*(*λ*) = *L*_1_(*λ*)(*Iλ* − *S*), then *Iλ* − *S* is said to be a monic right divisor of *L*(*λ*). As is well known, *Iλ* − *S* is a monic right divisor of *L*(*λ*) if and only if *S* is a root of *L*(*λ*); that is,

(2) $$\begin{array}{c} S^{l}+A_{l-1}S^{l-1}+\cdots +A_{0}=0. \end{array}$$

Let *C* be the companion matrix of *L*(*λ*); that is,

(3) $$\begin{array}{c} C=\left(\begin{array}{ccccc} 0 & I & 0 & \cdots & 0\\ 0 & 0 & I & \cdots & 0\\ \vdots & \vdots & \vdots & & \vdots \\ 0 & 0 & 0 & \cdots & I\\ -A_{0} & -A_{1} & -A_{2} & \cdots & -A_{l-1} \end{array}\right). \end{array}$$

Then we have det⁡(*Iλ* − *C*) = det⁡(*L*(*λ*)). If *S*_1_, *S*_2_,…, *S*_*l*_ ∈ *M*_*n*_(*C*) are the different *l* roots of *L*(*λ*), it is clear that the Vandermonde matrix

(4) $$\begin{array}{c} W\left(S_{1},S_{2},\ldots ,S_{l}\right)=\left(\begin{array}{ccccc} I & I & I & \cdots & I\\ S_{1} & S_{2} & S_{3} & \cdots & S_{l}\\ S_{1}^{2} & S_{2}^{2} & S_{3}^{2} & \cdots & S_{l}^{2}\\ \vdots & \vdots & \vdots & & \vdots \\ S_{1}^{l-1} & S_{2}^{l-1} & S_{3}^{l-1} & \cdots & S_{l}^{l-1} \end{array}\right) \end{array}$$

plays a role in the investigation of the spectral analysis of *L*(*λ*) [1].

**Definition 1**

We say that the *l*-tuple (*S*_1_, *S*_2_,…, *S*_*l*_) of complex square matrices of order *n* is complete if the Vandermonde matrix *W*(*S*_1_, *S*_2_,…, *S*_*l*_) is invertible. When *l* = 2 and *l* = 3, we call a complete *l*-tuple of matrices a complete pair and a complete triple, respectively.

Suppose that (*S*_1_, *S*_2_) is complete. Take *P* = *S*_2_ − *S*_1_. Then *P* is invertible. If we take

(5) A1=−PS2P−1−S1,  A2=PS2P−1S1,L(λ)=Iλ2+A1λ+A2,

then *Iλ* − *S*_1_, *Iλ* − *S*_2_, form a complete pair of right divisors of *L*(*λ*) (cf. Lemma 2.4 in [2]). The concept of complete pair was introduced in [3, 4]. In [5], Lancaster gave some important applications of complete pairs to solutions of differential equations. Motivated by the results in [5], we are interested in the following question.

**Question**

Suppose that (*S*_1_, *S*_2_) is not a complete pair. Is it possible for us to find *S*_3_ and a cubic matrix polynomial *L*(*λ*) such that (*S*_1_, *S*_2_, *S*_3_) is a complete triple and

(6) $$\begin{array}{c} I\lambda -S_{1},\;\;I\lambda -S_{2},\;\;I\lambda -S_{3} \end{array}$$

are right divisors of *L*(*λ*)?

If (*S*_1_, *S*_2_, *S*_3_) is a complete triple, then

(7) $$\begin{array}{c} \left(\begin{array}{cc} I & I\\ S_{1} & S_{2}\\ S_{1}^{2} & S_{2}^{2} \end{array}\right) \end{array}$$

is of full column rank. Note that if *S*_1_, *S*_2_ ∈ *M*_*n*_(*C*) and the rank of *S*_1_ − *S*_2_ is less than *n* − 1, we cannot always find *S*_3_ such that (*S*_1_, *S*_2_, *S*_3_) is a complete triple. For example, let *S*_1_, *S*_2_ ∈ *M*_*n*_(*C*) be defined as follows:

(8) $$\begin{array}{c} S_{1}=\left(\begin{array}{cc} A_{1} & \alpha \\ \beta & A_{2} \end{array}\right), \end{array}$$

where *A*_1_, *A*_2_ are square matrices of orders *n* − 2 and 2, respectively, and *S*_2_ − *S*_1_ is in the form

(9) $$\begin{array}{c} \left(\begin{array}{ccc} I_{n-2} & 0 & 0\\ 0 & 0 & 0\\ 0 & 0 & 0 \end{array}\right). \end{array}$$

Then

(10) $$\begin{array}{c} S_{2}^{2}-S_{1}^{2}=\left(\begin{array}{cc} 2A_{1}+I_{n-2} & \alpha \\ \beta & 0 \end{array}\right). \end{array}$$

If rank⁡(*α*) = 1, then the last two columns of $\left(\begin{array}{c} S_{2}-S_{1}\\ S_{2}^{2}-S_{1}^{2} \end{array}\right)$ are linearly dependent. For any *S*_3_ ∈ *M*_*n*_(*C*),

(11) $$\begin{array}{c} \left(\begin{array}{ccc} I & I & I\\ S_{1} & S_{2} & S_{3}\\ S_{1}^{2} & S_{2}^{2} & S_{3}^{2} \end{array}\right) \end{array}$$

cannot be invertible.

If rank⁡(*α*) = 2, then

(12) $$\begin{array}{c} \left(\begin{array}{cc} I & I\\ S_{1} & S_{2}\\ S_{1}^{2} & S_{2}^{2} \end{array}\right) \end{array}$$

is of full column rank. From the condition that

(13) $$\begin{array}{c} \left(\begin{array}{cc} I & I\\ S_{1} & S_{2}\\ S_{1}^{2} & S_{2}^{2} \end{array}\right) \end{array}$$

is of full column rank, we cannot conclude that rank⁡(*S*_2_ − *S*_1_) ≥ *n* − 1.

After the above discussion, we give the following definition.

**Definition 2**

For two matrices *S*_1_, *S*_2_ ∈ *M*_*n*_(*C*), one says they form an almost complete pair if the matrix

(14) $$\begin{array}{c} \left(\begin{array}{cc} I & I\\ S_{1} & S_{2}\\ S_{1}^{2} & S_{2}^{2} \end{array}\right) \end{array}$$

is of full column rank and the rank of *S*_1_ − *S*_2_ is *n* − 1. For simplification, one says (*S*_1_, *S*_2_) is an almost complete pair of matrices of order *n*.

The following is the main result of this paper.

**Theorem**

Suppose that *S*_1_, *S*_2_ ∈ *M*_*n*_(*C*), *n* ≥ 2, and (*S*_1_, *S*_2_) is an almost complete pair. Then one can find a matrix *S*_3_ ∈ *M*_*n*_(*C*) such that (*S*_1_, *S*_2_, *S*_3_) is a complete triple; that is,

(15) $$\begin{array}{c} \left(\begin{array}{ccc} I & I & I\\ S_{1} & S_{2} & S_{3}\\ S_{1}^{2} & S_{2}^{2} & S_{3}^{2} \end{array}\right) \end{array}$$

is invertible.

We prove this theorem in Section 2. In Section 3, we partially answer the question mentioned before and give an application of the main result to differential equations.

## 2. Proof of the Main Theorem 

In this section, we prove Theorem 3. Note that there is an invertible matrix *P* such that *P* ^−1^(*S*_2_ − *S*_1_)*P* is the Jordan normal form of *S*_2_ − *S*_1_. Let *Q* = *P* ⊕ *P* ⊕ *P*. Then the condition that

(16) $$\begin{array}{c} X=\left(\begin{array}{ccc} I & I & I\\ S_{1} & S_{2} & S_{3}\\ S_{1}^{2} & S_{2}^{2} & S_{3}^{2} \end{array}\right) \end{array}$$

is invertible is equivalent to that

(17) $$\begin{array}{c} Q^{-1}XQ \end{array}$$

is invertible. So from the beginning, we can assume that *S*_2_ − *S*_1_ is in Jordan normal form.

First we prove a lemma.

**Lemma**

Assume *S*_1_, *S*_2_ ∈ *M*_*n*_(*C*) and the pair (*S*_1_, *S*_2_) is an almost complete pair with *S*_2_ − *S*_1_ = *I*_*n*−1_ ⊕ 0. Write

(18) $$\begin{array}{c} S_{1}=\left(\begin{array}{cc} A & \alpha \\ \beta & s \end{array}\right), \end{array}$$

where *A* ∈ *M*_*n*−1_(*C*). Then *α* is not a zero vector.

**Proof**

By some computations, we have

(19) $$\begin{array}{c} S_{2}^{2}-S_{1}^{2}=\left(\begin{array}{cc} 2A+I_{n-1} & \alpha \\ \beta & 0 \end{array}\right). \end{array}$$

The last column of

(20) $$\begin{array}{c} \left(\begin{array}{c} S_{2}-S_{1}\\ S_{2}^{2}-S_{1}^{2} \end{array}\right) \end{array}$$

is (0,0,…, 0, *α*, 0)^*T*^, which is a 2*n*-dimensional column vector. By the definition of almost complete pair, *α* is not a zero vector.

Now we prove the main theorem.

**Proof**

From now on, we write S1=(a11⋯a1n⋮⋱⋮an1⋯ann). We denote by *P*(*i*, *j*) ∈ *M*_*n*−1_(*R*) the matrix which is obtained by exchanging the columns of *i* and *j* of (*n* − 1)×(*n* − 1) identity matrix.

Without loss of generality, we can assume *S*_2_ − *S*_1_ is in the Jordan normal form.

*Case  1*. Firstly, we consider the case that

(21) $$\begin{array}{c} S_{2}-S_{1}=\left(\begin{array}{cc} I_{n-1} & 0\\ 0 & 0 \end{array}\right). \end{array}$$

If *a*_1*n*_ ≠ 0, then we take

(22) $$\begin{array}{c} S_{3}=S_{1}+\left(\begin{array}{cc} 0 & 0\\ tI_{n-1} & 0 \end{array}\right). \end{array}$$

By some computation, the last columns of *S*_2_^2^ − *S*_1_^2^ and *S*_3_^2^ − *S*_1_^2^ are (*a*_1*n*_, *a*_2*n*_,…, *a*_*n*−1  *n*_, 0)^*T*^ and (0, *ta*_1*n*_, *ta*_2*n*_,…, *ta*_*n*−1  *n*_)^*T*^, respectively, and the *i*th (1 ≤ *i* ≤ *n* − 2) column of *S*_3_^2^ − *S*_1_^2^ is

(23) (fi,1(t),fi,2(t),…,fi,i+1(t),t2+fi,i+2(t),fi,i+3(t),…,fi,n(t))T,

and the (*n* − 1)th column of *S*_3_^2^ − *S*_1_^2^ is (*f*_*n*−1,1_(*t*), *f*_*n*−1,2_(*t*),…, *f*_*n*−1, *n*_(*t*))^*T*^, where *f*_*i*,1_ = *ta*_1  *i*+1_ and *f*_*i*,*j*_ = *t*(*a*_*j* *i*+1_ + *a*_*j*−1  *i*_)(1 ≤ *i* ≤ *n* − 1,  2 ≤ *j* ≤ *n*). It is clear that

(24) $$\begin{array}{c} \det\left(\begin{array}{cc} S_{2}-S_{1} & S_{3}-S_{1}\\ S_{2}^{2}-S_{1}^{2} & S_{3}^{2}-S_{1}^{2} \end{array}\right) \end{array}$$

is a polynomial in *t*. If *a*_1*n*_ ≠ 0, then the degree of this polynomial is 2*n* − 2 and the leading term of this polynomial is *a*_1*n*_^2^ *t* ^2*n*−2^. If |*t*| is sufficiently large, we can get that

(25) $$\begin{array}{c} X=\left(\begin{array}{ccc} I & I & I\\ S_{1} & S_{2} & S_{3}\\ S_{1}^{2} & S_{2}^{2} & S_{3}^{2} \end{array}\right) \end{array}$$

is invertible. If *a*_1*n*_ = 0, by Lemma 4, then there must be some

(26) $$\begin{array}{c} a_{in}\neq 0, \end{array}$$

where 2 ≤ *i* ≤ *n* − 1. So

(27) (P(1,i)001)(Aαβ0)(P(1,i)001) =((P(1,i))AP(1,i)P(1,i)αβP0),

where the first entry of the vector *P*(1, *i*)*α* is *a*_*in*_. Take *S*_3_ such that

(28) $$\begin{array}{c} \left(P\left(1,i\right)⊕1\right)\left(S_{3}-S_{1}\right)\left(P\left(1,i\right)⊕1\right)=\left(\begin{array}{cc} 0 & 0\\ tI_{n-1} & 0 \end{array}\right). \end{array}$$

Then

(29) $$\begin{array}{c} X=\left(\begin{array}{ccc} I & I & I\\ S_{1} & S_{2} & S_{3}\\ S_{1}^{2} & S_{2}^{2} & S_{3}^{2} \end{array}\right) \end{array}$$

is invertible.

*Case  2*. Now we consider the case that *S*_2_ − *S*_1_ has the Jordan normal form

(30) $$\begin{array}{c} J\left(\lambda_{1}\right)⊕J\left(\lambda_{2}\right)⊕\cdots ⊕J\left(\lambda_{k}\right)⊕0, \end{array}$$

where *λ*_*i*_ ≠ 0, 1 ≤ *i* ≤ *k*. If *a*_1*n*_ ≠ 0, then we take

(31) $$\begin{array}{c} S_{3}=S_{1}+\left(\begin{array}{cc} 0 & 0\\ tI_{n-1} & 0 \end{array}\right). \end{array}$$

If *a*_1*n*_ = 0 and *a*_*in*_ ≠ 0  (1 < *i* < *n*), then we take *S*_3_ such that

(32) $$\begin{array}{c} \left(P\left(1,i\right)⊕1\right)\left(S_{3}-S_{1}\right)\left(P\left(1,i\right)⊕1\right)=\left(\begin{array}{cc} 0 & 0\\ tI_{n-1} & 0 \end{array}\right). \end{array}$$

In any case when |*t*| is sufficiently large, we can get that

(33) $$\begin{array}{c} X=\left(\begin{array}{ccc} I & I & I\\ S_{1} & S_{2} & S_{3}\\ S_{1}^{2} & S_{2}^{2} & S_{3}^{2} \end{array}\right) \end{array}$$

is invertible.

*Case  3*. Now we consider the case that 0 is not a single root of characteristic polynomial of *S*_2_ − *S*_1_; that is, *S*_2_ − *S*_1_ has the Jordan normal form:

(34) $$\begin{array}{c} J\left(\lambda_{1}\right)⊕J\left(\lambda_{2}\right)⊕\cdots ⊕J\left(\lambda_{s}\right)⊕J\left(0\right). \end{array}$$

If *n* = 2, then

(35) $$\begin{array}{c} S_{2}-S_{1}=\left(\begin{array}{cc} 0 & 0\\ 1 & 0 \end{array}\right), \end{array}$$

By Lemma 4, *a*_12_ ≠ 0. Take

(36) $$\begin{array}{c} S_{3}=S_{1}+\left(\begin{array}{cc} 1 & 0\\ 0 & 0 \end{array}\right), \end{array}$$

Then

(37) $$\begin{array}{c} \det\left(\begin{array}{ccc} I & I & I\\ S_{1} & S_{2} & S_{3}\\ S_{1}^{2} & S_{2}^{2} & S_{3}^{2} \end{array}\right)=\det\left(\begin{array}{cc} S_{2}-S_{1} & S_{3}-S_{1}\\ S_{2}^{2}-S_{1}^{2} & S_{3}^{2}-S_{1}^{2} \end{array}\right)=-t^{2}a_{12}^{2}\neq 0. \end{array}$$

We can conclude that

(38) $$\begin{array}{c} X=\left(\begin{array}{ccc} I & I & I\\ S_{1} & S_{2} & S_{3}\\ S_{1}^{2} & S_{2}^{2} & S_{3}^{2} \end{array}\right) \end{array}$$

is invertible.

Firstly, we prove the case that the normal form itself is

(39) $$\begin{array}{c} \left(\begin{array}{cc} 0 & 0\\ tI_{n-1} & 0 \end{array}\right). \end{array}$$

If *a*_1*n*_ ≠ 0, then we take

(40) $$\begin{array}{c} S_{3}=S_{1}+\left(\begin{array}{ccccccc} t & 0 & 0 & 0 & \cdots & 0 & t\\ 0 & 0 & 0 & 0 & \cdots & 0 & 0\\ t & t & 0 & 0 & \cdots & 0 & 0\\ 0 & 0 & t & 0 & \cdots & 0 & 0\\ \vdots & \vdots & \vdots & \ddots & & \vdots & \vdots \\ 0 & 0 & 0 & 0 & \cdots & t & 0 \end{array}\right). \end{array}$$

So

(41) $$\begin{array}{c} {\left(S_{3}-S_{1}\right)}^{2}=\left(\begin{array}{ccccccc} t^{2} & 0 & 0 & \cdots & 0 & t^{2} & 0\\ 0 & 0 & 0 & \cdots & 0 & 0 & 0\\ t^{2} & 0 & 0 & \cdots & 0 & 0 & 0\\ 0 & t^{2} & 0 & \cdots & 0 & 0 & 0\\ \vdots & \vdots & \ddots & & \vdots & \vdots & \vdots \\ 0 & 0 & 0 & \cdots & t^{2} & 0 & 0 \end{array}\right). \end{array}$$

By some computation,

(42) $$\begin{array}{c} \det X=\det\left(\begin{array}{ccc} I & I & I\\ S_{1} & S_{2} & S_{3}\\ S_{1}^{2} & S_{2}^{2} & S_{3}^{2} \end{array}\right) \end{array}$$

is a polynomial in *t*, where the leading term of it is *a*_1*n*_ *t* ^2*n*−1^. So if *a*_1*n*_ ≠ 0, then *X* is invertible when |*t*| is sufficiently large. If *a*_1*n*_ = 0, then there must be an *i* ∈ {2,3,…, *n* − 1} such that *a*_*in*_ ≠ 0. If *i* ∈ {2,3,…, *n* − 2}, take *S*_3_ such that

(43) (P(1,i−1)⊕1)(S3−S1)(P(1,i−1)⊕1) =(t000⋯0t0000⋯00tt00⋯0000t0⋯00⋮⋮⋮⋱⋮⋮0000⋯t0).

When |*t*| is sufficiently large, we have *X* being invertible. If *i* = *n* − 1, that is to say, the last column of *S*_2_^2^ − *S*_1_^2^ is (0,0,…, 0, *a*_*n*−1  *n*_)^*T*^, then we take

(44) $$\begin{array}{c} S_{3}=S_{1}+\left(\begin{array}{ccccccc} t & 0 & 0 & 0 & \cdots & 1 & 0\\ 0 & t & 0 & 0 & \cdots & 0 & 0\\ 0 & 0 & t & 0 & \cdots & 0 & 0\\ 0 & 0 & 0 & t & \cdots & 0 & 0\\ \vdots & \vdots & \vdots & \vdots & \ddots & \vdots & \vdots \\ 0 & 0 & 0 & 0 & \cdots & t & 0\\ 0 & 0 & 0 & 0 & \cdots & 0 & 0 \end{array}\right). \end{array}$$

We have

(45) $$\begin{array}{c} {\left(S_{3}-S_{1}\right)}^{2}=\left(\begin{array}{ccccccc} t^{2} & 0 & 0 & 0 & \cdots & 2t & 0\\ 0 & t^{2} & 0 & 0 & \cdots & 0 & 0\\ 0 & 0 & t^{2} & 0 & \cdots & 0 & 0\\ 0 & 0 & 0 & t^{2} & \cdots & 0 & 0\\ \vdots & \vdots & \vdots & \vdots & \ddots & \vdots & \vdots \\ 0 & 0 & 0 & 0 & \cdots & t^{2} & 0\\ 0 & 0 & 0 & 0 & \cdots & 0 & 0 \end{array}\right), \end{array}$$

where the first entry of the last column of *S*_3_^2^ − *S*_1_^2^ is *a*_*n*−1  *n*_. So the leading term of the polynomial

(46) $$\begin{array}{c} \det X=\det\left(\begin{array}{ccc} I & I & I\\ S_{1} & S_{2} & S_{3}\\ S_{1}^{2} & S_{2}^{2} & S_{3}^{2} \end{array}\right) \end{array}$$

is *a*_*n*−1  *n*_^2^ *t* ^2*n*−3^. Thus

(47) $$\begin{array}{c} X=\left(\begin{array}{ccc} I & I & I\\ S_{1} & S_{2} & S_{3}\\ S_{1}^{2} & S_{2}^{2} & S_{3}^{2} \end{array}\right) \end{array}$$

is invertible when |*t*| is sufficiently large.

Now we prove the general case that *S*_2_ − *S*_1_ has the Jordan normal form:

(48) $$\begin{array}{c} J\left(\lambda_{1}\right)⊕J\left(\lambda_{2}\right)⊕\cdots ⊕J\left(\lambda_{s}\right)⊕J\left(0\right), \end{array}$$

where *λ*_*i*_ ≠ 0, 1 ≤ *i* ≤ *s*. If there is some *a*_*ni*_ ≠ 0, *m* + 1 ≤ *i* ≤ *n*, we assume that *J*(0) is a matrix of order *n* − *m*. For any square matrix *B* of order *n* − *m*, we can construct a square matrix *A* of order *n* − *m* such that

(49) $$\begin{array}{c} \left(\begin{array}{cc} J\left(0\right) & A\\ {\left(J(0)+B\right)}^{2}-J^{2}\left(0\right) & {\left(A+B\right)}^{2}-A^{2} \end{array}\right) \end{array}$$

is invertible. Take

(50) $$\begin{array}{c} S_{3}=S_{1}+\left(tI_{n-m}⊕A\right). \end{array}$$

If *a*_*in*_ = 0, for all *m* + 1 ≤ *i* ≤ *n*, there must be some *j* such that 1 ≤ *j* ≤ *m*, *a*_*jn*_ ≠ 0. Let *Y* = *XP*, where *P* is the matrix which is obtained by exchanging the columns *m* + 1 and *n* of *n* × *n* identity matrix. Then det⁡*X* = −det⁡*Y* and

(51) $$\begin{array}{c} Y=J\left(\lambda_{1}\right)⊕J\left(\lambda_{2}\right)⊕\cdots ⊕J\left(\lambda_{i}\right)⊕0⊕C, \end{array}$$

where the square matrix *C* of order *n* − *m* + 1 has the form

(52) $$\begin{array}{c} \left(\begin{array}{cc} 0 & 1\\ I_{n-m} & 0 \end{array}\right). \end{array}$$

Let *T* be a matrix such that *T* ^−1^ *CT* has the Jordan normal form *J*. Then we can consider the matrix *Z* = (*I* ⊕ *T* ^−1^)*Y*(*I* ⊕ *T*). Note that *Z* has the form

(53) $$\begin{array}{c} Z=J\left(\lambda_{1}\right)⊕J\left(\lambda_{2}\right)⊕\cdots ⊕J\left(\lambda_{i}\right)⊕0⊕J. \end{array}$$

Denote *S* = *J*(*λ*_1_) ⊕ *J*(*λ*_2_)⊕⋯⊕*J*(*λ*_*i*_) ⊕ 0. For any square matrix *B*, we can construct a square matrix *A* of order *m* + 1 such that

(54) $$\begin{array}{c} \left(\begin{array}{cc} S & A\\ {\left(S+B\right)}^{2}-S^{2} & {\left(A+B\right)}^{2}-A^{2} \end{array}\right) \end{array}$$

is invertible. Take *S*_3_ = *S*_1_ + *A* ⊕ *tI*_*m*−1_. Then we can conclude that

(55) $$\begin{array}{c} \left(\begin{array}{ccc} I & I & I\\ S_{1} & S_{2} & S_{3}\\ S_{1}^{2} & S_{2}^{2} & S_{3}^{2} \end{array}\right) \end{array}$$

is invertible. The proof is complete.

## 3. Applications 

For given monic matrix polynomials *L*_1_(*λ*),  *L*_2_(*λ*),  …,  *L*_*n*_(*λ*), the construction of a common multiple of them was presented in [2].

Suppose that (*S*_1_, *S*_2_) is an almost complete pair. By Theorem 3 in Section 1, we can find *S*_3_ ∈ *M*_*n*_(*C*) such that (*S*_1_, *S*_2_, *S*_3_) is a complete triple; that is,

(56) $$\begin{array}{c} \left(\begin{array}{ccc} I & I & I\\ S_{1} & S_{2} & S_{3}\\ S_{1}^{2} & S_{2}^{2} & S_{3}^{2} \end{array}\right) \end{array}$$

is invertible. Let

(57) $$\begin{array}{c} W_{m}\left(S_{1},S_{2},S_{3}\right)=\left(\begin{array}{ccc} I & I & I\\ S_{1} & S_{2} & S_{3}\\ \vdots & \vdots & \vdots \\ S_{1}^{m-1} & S_{2}^{m-1} & S_{3}^{m-1} \end{array}\right). \end{array}$$

Then we can conclude that

(58) $$\begin{array}{c} \min\left\{m\geq 1\;|\;\mathrm{Ker}W_{m}=\mathrm{Ker}W_{m+1}\right\}=3. \end{array}$$

By Theorem 9.11 of [2], there exists a monic matrix polynomial *L*(*λ*) with degree 3 which is a common multiple of *Iλ* − *S*_1_, *Iλ* − *S*_2_, *Iλ* − *S*_3_.

As is well known, spectral method for the analysis of monic matrix polynomials was important (cf. [6–13]). The spectral method basically uses the construction of standard pairs and standard triples (e.g., [14, 15]) to solve differential equations.

Now we recall definitions of standard pair and standard triple for monic polynomials.

**Definition 5 (see [2])**

A pair of matrices (*X*, *T*), where *X* is *n* × *nl* and *T* is *nl* × *nl*, is called a standard pair for the monic matrix polynomial *L*(*λ*) if the following conditions are satisfied:

(i) col(*XT* ^*i*^)_*i*=0_^*l*−1^ is nonsingular, where
  (59) $$\begin{array}{c} \text{col}{\left(XT^{i}\right)}_{i=0}^{l-1}=\left(\begin{array}{c} X\\ XT\\ \vdots \\ XT^{l-1} \end{array}\right); \end{array}$$
(ii) Σ_*i*=0_^*l*−1^ *A*_*i*_ *XT* ^*i*^ + *XT* ^*l*^ = 0.

If *T* is in Jordan normal form, we call (*X*, *T*) a Jordan pair for the monic matrix polynomial *L*(*λ*).

**Definition 6 (see [2])**

A triple of matrices (*X*, *T*, *Y*), where *X* is *n* × *nl*, *T* is *nl* × *nl*, and *Y* is *nl* × *n*, is called a standard triple for the monic matrix polynomial *L*(*λ*) if (*X*, *T*) is a standard pair for *L*(*λ*), and Y=(XXT⋮XTl-1)-1(0⋮0I).

Now we recall the explicit formulas for solutions of the following differential equation:

(60) $$\begin{array}{c} L\left(\frac{d}{dt}\right)x\left(t\right)=\frac{d^{l}x\left(t\right)}{dt^{l}}+\Sigma_{j=0}^{l-1}A_{j}\frac{d^{j}x\left(t\right)}{dt^{j}}=f\left(t\right). \end{array}$$

**Lemma 7 (Theorem 2.9 in [2])**

The general solution of (60) is given by the formula

(61) $$\begin{array}{c} x\left(t\right)=Xe^{tT}c+X\overset{t}{\underset{a}{\int }}e^{(t-s)T}Yf\left(s\right)ds,\;t\in \left[a,b\right], \end{array}$$

where (*X*, *T*, *Y*) is a standard triple of *L*(*λ*) and *c* ∈ *C* ^*nl*^ is arbitrary. In particular, the general solution of the homogeneous equation

(62) $$\begin{array}{c} L\left(\frac{d}{dt}\right)x\left(t\right)=0 \end{array}$$

is given by the formula

(63) $$\begin{array}{c} x\left(t\right)=Xe^{tT}c,\;c\in C^{nl}. \end{array}$$

Before we give the main result of this section, we introduce some notations. Suppose

(64) $$\begin{array}{c} J_{1}=X_{1}^{-1}S_{1}X_{1},\;\;J_{2}=X_{2}^{-1}S_{2}X_{2},\;\;J_{3}=X_{3}^{-1}S_{3}X_{3} \end{array}$$

are the Jordan forms of *S*_1_, *S*_2_, *S*_3_, respectively. Write

(65) $$\begin{array}{c} X=\left(\begin{array}{ccc} X_{1} & X_{2} & X_{3} \end{array}\right),\;\;J=J_{1}⊕J_{2}⊕J_{3}. \end{array}$$

**Theorem 8**

Let (*S*_1_, *S*_2_, *S*_3_) be a complete triple of *L*(*λ*) = 0, where *L*(*λ*) = *Iλ* ^3^ + *A*_2_ *λ* ^2^ + *A*_1_ *λ* + *A*_0_. Then every solution of

(66) $$\begin{array}{c} L\left(\frac{d}{dt}\right)u=f \end{array}$$

is given by

(67) u(t)=eS1tc1+eS2tc2+eS3tc3 +X∫ate(t−s)J(Y1Y2Y3)f(s)ds, t∈[a,b],

for some *c*_1_, *c*_2_, *c*_3_ ∈ *C* ^*n*^, where

(68) (Y2Y3)=(S2−S1S3−S1S22−S12S32−S12)−1(0X3−1),Y1=−X1−1X2Y2−X1−1X3Y3.

In particular, every solution of

(69) $$\begin{array}{c} L\left(\frac{d}{dt}\right)u=0 \end{array}$$

has the form

(70) $$\begin{array}{c} u\left(t\right)=e^{S_{1}t}c_{1}+e^{S_{2}t}c_{2}+e^{S_{3}t}c_{3}, \end{array}$$

where *c*_1_, *c*_2_, *c*_3_ ∈ *C* ^*n*^.

**Proof**

Using Lemma 7, we take

(71) $$\begin{array}{c} Y=\left(\begin{array}{c} Y_{1}\\ Y_{2}\\ Y_{3} \end{array}\right)=X^{-1}{\left(\begin{array}{ccc} I & I & I\\ S_{1} & S_{2} & S_{3}\\ S_{1}^{2} & S_{2}^{2} & S_{3}^{2} \end{array}\right)}^{-1}\left(\begin{array}{c} 0\\ 0\\ I \end{array}\right). \end{array}$$

Then (*X*, *J*, *Y*) is a standard triple; it is easy to get the conclusion.

**Remark 9**

The above theorem and its proof are motivated by those in [5] (cf. also Sections 2.4 and 2.5 in [2]).
